# Surgical pathology and the diagnosis of invasive visceral yeast infection: two case reports and literature review

**DOI:** 10.1186/1749-7922-8-38

**Published:** 2013-09-26

**Authors:** Paola Di Carlo, Gaetano Di Vita, Giuliana Guadagnino, Gianfranco Cocorullo, Francesco D’Arpa, Giuseppe Salamone, Buscemi Salvatore, Gaspare Gulotta, Daniela Cabibi

**Affiliations:** 1 Department of Sciences for Health Promotion and Mother-Child Care “G. D’Alessandro”, University of Palermo, Via del Vespro 127, I- 90127 Palermo, Italy; 2 Department of Surgery and Oncology Sciences, General Surgery Unit, University of Study of Palermo, Italy, Via del Vespro 127, I- 90127 Palermo, Italy; 3 Department of General Surgery, Urgency and Organ Transplantation, University of Palermo, Via del Vespro 127, I- 90127 Palermo, Italy

**Keywords:** Surgical pathology, Gastrointestinal candidiasis, Diagnosis

## Abstract

Invasive mycoses are life-threatening opportunistic infections that have recently emerged as a cause of morbidity and mortality following general and gastrointestinal surgery.

*Candida* species are the main fungal strains of gut flora. Gastrointestinal tract surgery might lead to mucosal disruption and cause *Candida* spp. to disseminate in the bloodstream.

Here we report and discuss the peculiar clinical and morphological presentation of two cases of gastrointestinal C*andida albicans* lesions in patients who underwent abdominal surgery.

Although in the majority of cases reported in the literature, diagnosis was made on the basis of microbiological criteria, we suggest that morphological features of fungi in histological sections of appropriate surgical specimens could help to detect the degree of yeast colonization and identify patients at risk of developing severe abdominal *Candida* infection.

Better prevention and early antifungal treatments are highlighted, and relevant scientific literature is reviewed.

## Introduction

Invasive mycoses are important healthcare-associated infections, and have become an increasingly frequent problem in immunocompromised and severely ill patients [1]. Medical progress, which has resulted in a growing number of invasive procedures, new dimensions in aggressive immunosuppressive and immunomodulatory treatments and widespread use of broad-spectrum antibiotics, is the main catalyst for this development [1-3].

Invasive fungal infections, *Candida* species in particular, are the fourth most common cause of nosocomial bloodstream infections, and are associated with high morbidity and mortality in critically-ill patients, particularly those who have recently undergone extensive gastro-abdominal surgery [4].

Several studies conducted over the last two decades have shown that gastrointestinal surgeries are associated with an increased risk of fungemia, and patients admitted to surgical intensive care units (ICUs) are considered to have a greater risk of developing it [3,4].

*Candida* spp. are the main fungal strains of gut flora. Gastrointestinal tract surgery might lead to mucosal disruption and cause *Candida* spp. to disseminate through the bloodstream.

Lastly, despite a strong index of suspicion in high-risk subjects such as patients who require surgical re-intervention, and international guidelines on the use of antifungal prophylaxis, the incidence and severity of candidiasis in post-surgical patients appears significant. Moreover, isolated species show virulence factors and exhibit varying levels of susceptibility to antifungal drugs [1,5,6].

In the present study, we report two cases of *Candida albicans* infection identified in abdominal specimens from patients who had undergone gastro-abdominal surgery.

## Case presentation

### First case

In December 2012, a 54 year-old woman of Italian origin and nationality presented to the general surgery and emergency unit of the “P. Giaccone” Teaching Hospital in Palermo, Italy, with severe epigastric left-upper-quadrant pain that was progressive and burning.

Her medical history was significant for hypertension, asthma and rectal cancer surgery (T_1_N_0_M_0_) involving low anterior resection with total mesorectal excision and end to end anastomosis in October 2012. Recovery from surgery was hampered by recurrent episodes of fever but no specific infectious agent was detected; in view of this, the patient showed clinical improvement after empirical treatment with fluconazole.

Physical examination revealed a soft abdomen with positive bowel sounds, and tenderness to palpation in the left upper quadrant. Rectal examination was guaiac-negative, and a complete blood count indicated leukocytosis with left shift. CT scan of abdomen showed a gastric dilatation, marked thickening of the anterior wall and necrotic areas within.

An exploratory upper laparotomy confirmed acute gastric dilatation and necrosis of the anterior surface of the stomach. A “sleeve” gastrectomy to ablate the necrotic area was performed and a feeding jejunostomy.

**Figure 1:**
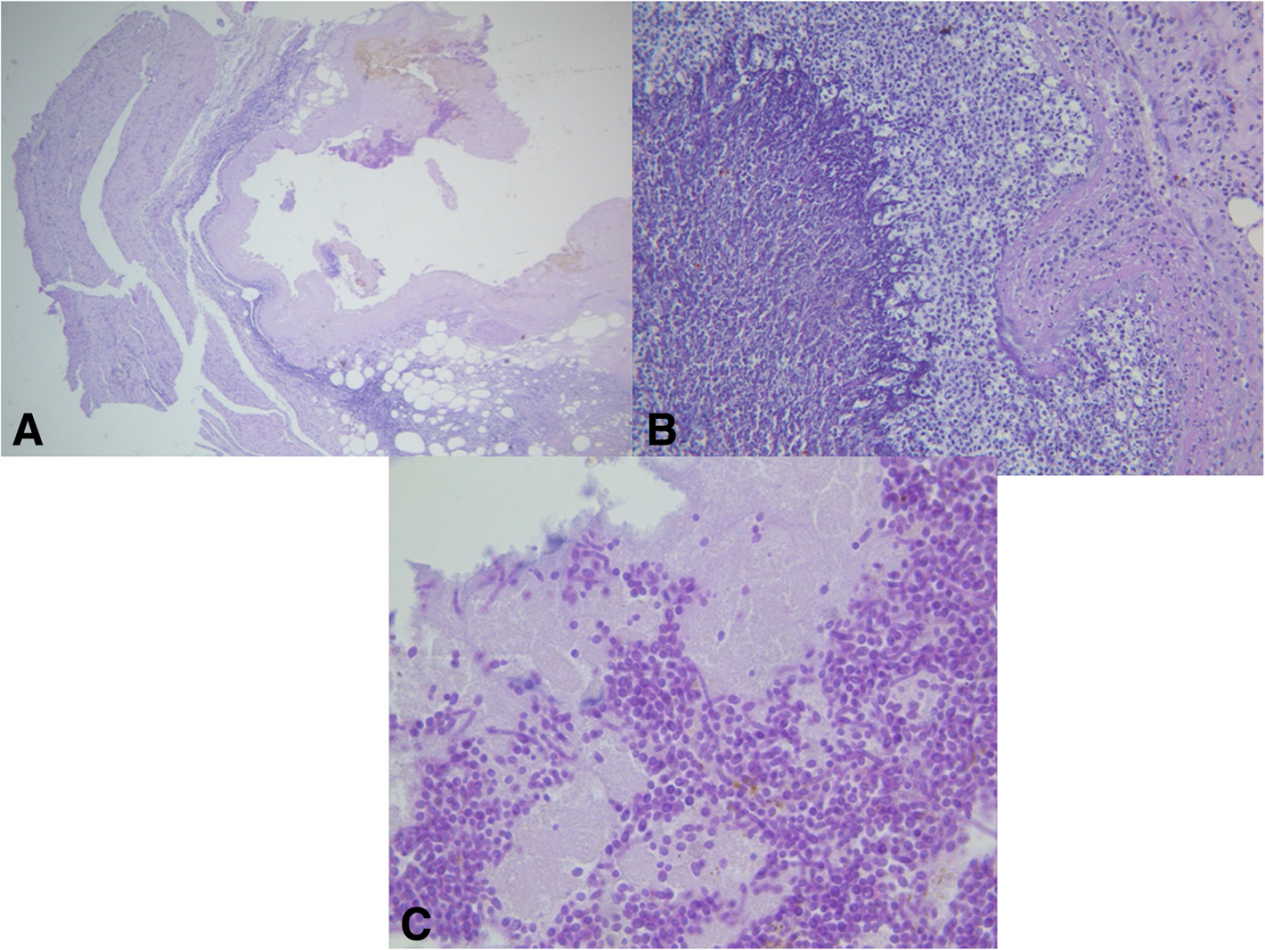
**Histological section. A)** Very thin and totally necrotic gastric wall. **B, C)** Numerous fungal yeasts were present. PAS stain **(A)** ×100; **(B)** ×200; **(C)** ×400.

The gastric wall appeared very thin and totally necrotic upon macroscopic examination by the pathologist. No layers or structures were identifiable on histological examination, but numerous fungal yeasts were identified inside the necrotic areas with PAS and Gomori Silvermthenamina stains (Figure 1).

Culture of the intra-operative surgical specimen confirmed the presence of *Candida albicans*.

Yeast isolates were identified to the species level by conventional morphological and biochemical methods, as previously reported [3,7,8].

The yeast isolate was susceptible to fluconazole and echinocandin, according to CLSI cut off values [9,10]. It is noteworthy that blood cultures were negative.

Echinocandin (70 mg on the first day, i.e., day 103, followed by 50 mg/day) was administered parenterally for a total of 14 days, followed by maintenance therapy with 400 mg of oral fluconazole per day.

The patient was discharged in stable condition and antifungal therapy was continued in an outpatient setting. She has been doing well since then.

### Second case

In January 2013, a 62 year-old woman of Italian origin and nationality with BMI of 35 kg/m^2^, presented to the general surgery and emergency unit of the “P. Giaccone” Teaching Hospital in Palermo, Italy, with complicated midline incisional hernia, nausea, vomiting and abdominal distension.

Her initial vital signs were notable for a temperature of 38°C, respiratory rate of 22 breaths per minute, heart rate of 110 beats per minute and blood pressure of 90/60 mmHg. She was suffering from severe abdominal pain and breathing difficulties. On clinical examination, she presented a tender abdomen, ulcerated skin with associated necrosis and dry skin.

Her past medical history showed three caesarean sections, treatment for arterial hypertension, COPD and a diagnosis of type II diabetes mellitus (DM) about 15 years previously, treated with insulin.

Emergency surgery was required, and surgical exploration showed a congested, edematous and necrotic strangulated intestinal tract. The section of necrotic intestine was removed and ileo-ileostomy was performed. The surgery was successful, without additional complications, and an abdominal subcutaneous drain was inserted. The surgical specimen was sent to the Pathology Laboratory for histological examination.

During the postoperative period, the patient remained febrile but two routine blood cultures were negative.

**Figure 2:**
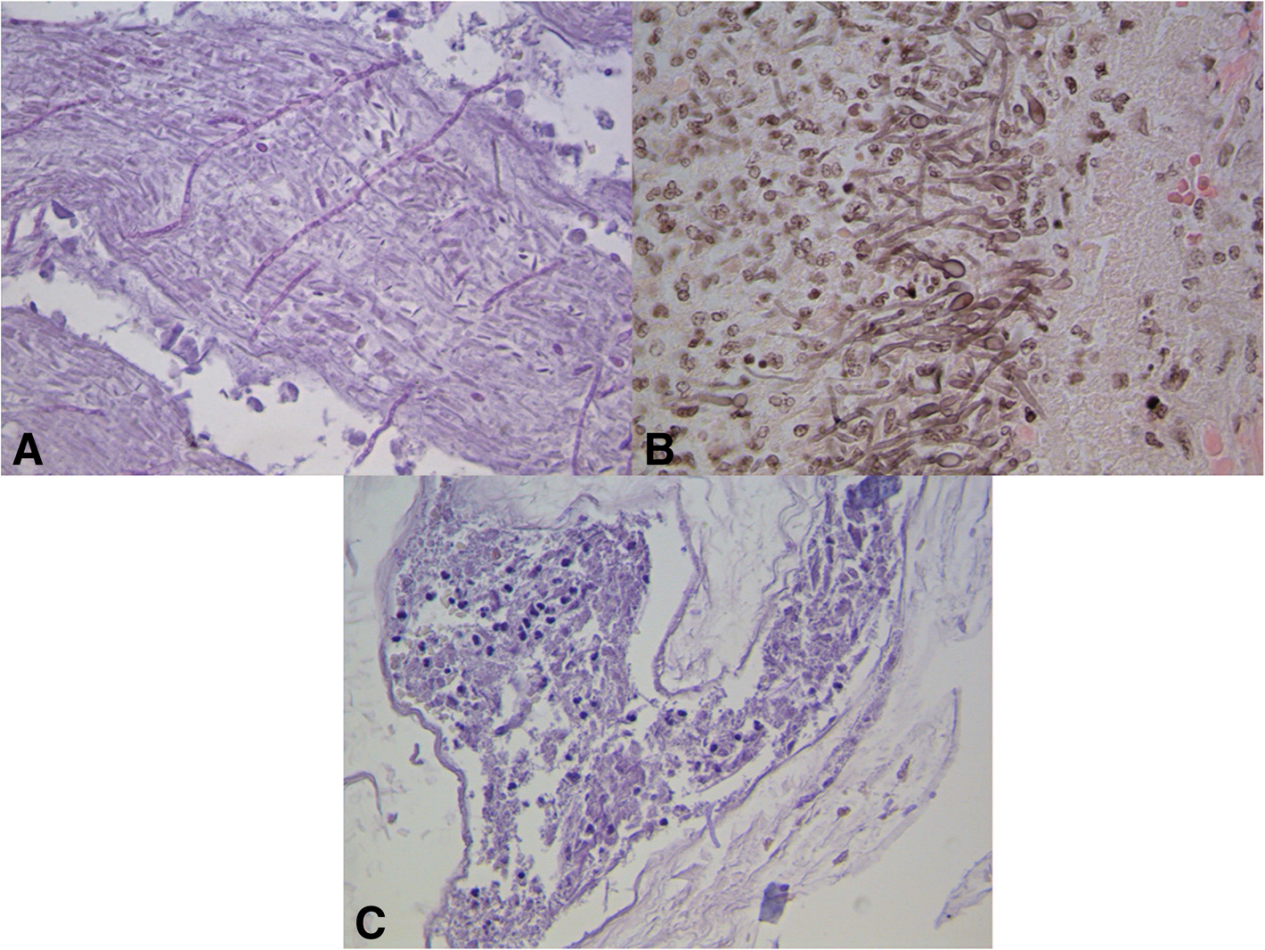
**Histological section. A)** Necrotic tissue from the cutaneous specimen, with fungal hyphae. **B-C)** Hyphae in the small bowel specimen. In **C** some of them appear to cross the vessel wall. PAS stain **(A)** ×200; GMS stain **(B)** ×400, PAS stain **(C)** ×200.

Furthermore, histological examination of both the skin and the small bowel specimens using special histochemical stains (PAS, Gomori Silvermethenamine) showed severe inflammation and massive areas of necrosis containing fungal spores and numerous budding hyphae (Figure 2).

Some yeasts were present across vessel walls of the small bowel, suggesting systemic blood dissemination (Figure 2C).

These findings were in keeping with culture results of intraoperative specimens and serial drainage fluids, showing fluconazole-resistant *Candida albicans*, susceptible to echinocandin according to CLSI cut off values [8].

Echinocandin (70 mg on the first day, i.e., day 103, followed by 50 mg/day) was administered parenterally for a total of 21 days.

The patient’s clinical conditions improved, fever disappeared and she was subsequently discharged in a good clinical state.

## Discussion

We have reported two cases of abdominal surgery patients who developed systemic candidiasis, and whose clinical symptoms improved following the initiation of therapy with 70/50 mg/day echinocandin. Oral thrush and esophageal candidiasis are the most common manifestations of Candida infection in the GI tract, with only occasional involvement of the colon and rectum. Despite the high concentration of *Candida* spp. in the lower GI tract, infection does not occur under normal circumstances, owing to innate defense mechanisms.

In this manuscript, we have described abdominal lesions due to *Candida albicans* infection. In a previous case report, we described a vegetating gastric *Candida albicans* lesion in an immunocompetent patient, endoscopically simulating a neoplasia [11]. This study reports two new cases of abdominal fungal infection in patients who had undergone abdominal surgery.

Gastrointestinal candida lesions remain difficult to diagnose because of the prevalence of colonization without accompanying infection, non-specific symptoms, and variable presentation.

In our two cases, despite blood cultures being negative for yeast, the histological analysis, performed with special histochemical stains, and culture of specimens or drainage fluid allowed us to identify it.

Although new, rapid and sensitive methods for diagnosing invasive fungal disease are available [12], histopathologic examination remains one of the major diagnostic tools in mycology because it permits rapid, presumptive identification of fungal infections [13,14].

Newer fungal, invasive visceral candidiasis and multidrug-resistant bacteria involving hollow gastrointestinal viscera are emerging pathologies for abdominal surgery [11,14,15].

Minali et al. reported that stomach candidiasis was seen in 0.96% of upper intestinal endoscopies [16]. Furthermore, Gupta described a fatal case of gastric perforation where biopsy revealed fungal hyphae [17].

In our patients’ case, systemic echinocandin treatment led to a substantial improvement in their clinical condition and a favorable outcome, despite the presence of several risk factors such as diabetes and/or re-intervention.

In the first case, fever disappeared with antifungal treatment after rectal cancer surgery was suggestive of fungemia and the authors hypothesized that fungal flora which normally colonize the gastric mucosa may overgrow under certain conditions, resulting in mucosal lesions of the digestive tract, in different sites and regardless of the site of surgery [18].

In fact, our patient had undergone rectal resection for neoplasm, but surgical re-intervention was required after 2 months for necrosis of the stomach, when diffuse yeasts were observed.

In the second case, where the reason for surgery was a complicated incisional hernia, we believe that the presence of candida was due to a superinfection in intestinal tissue which had undergone necrotic degeneration due to mechanical reasons. However, the cutaneous abscess adjacent to the complicated incisional hernia where numerous hyphae were also observed, could be a primary abscess due to disseminated intestinal fungal overgrowth. In both cases, early detection of *Candida albicans* by culture and histology permitted us to start the correct therapeutic approach with echinocandin, which led to a rapid improvement in the patients’ clinical condition.

In one study by Ears et al., gut mycosis was observed in 109 (4.35%) out of 2517 cases studied from 1960–1964 [19].

In Japan, Tsukamoto et al. reported that gut mycosis was present in 196 (5.9%) out of 3,339 cases recorded from 1971 to 1983 [20]. In these reports, the most commonly-affected organ was the esophagus, followed by the stomach, the small intestine and the large intestine [17,20].

Although the presence of *Candida* spp.in intra-abdominal specimens is associated with increased mortality in certain subgroups of patients, both of our patients with *Candida albicans* involvement had a favorable outcome after echinocandin treatment.

The use of Echinocandins is justified by their poor ability to develop resistance, which makes these molecules notably effective and reliable. Current guidelines recommend them for the treatment of targeted candidemia [2,6,21].

Previous studies have demonstrated the importance of echinocandin in patients who have recently undergone abdominal surgery, who present recurrent gastrointestinal perforations, anastomotic failure, are ventilated, hospitalized for more than 3 days, treated with broad-spectrum antibiotics and who have a CVC inserted [22,23].

Further studies are needed to define the sensitivity and specificity of this assay to diagnose fungal infection prior to the existence of other clinical or laboratory indications of invasive fungal infection.

## Conclusions

Three interesting findings emerge from these case reports. Firstly, the clinical recognition and effective management of fungal infections in surgical settings is challenging and the strategy to reduce damages needs a multistep diagnostic approach to establish a certain diagnosis.

Secondly, the study underlines the importance of culture and histological examination of surgical specimens, which could detect the presence of fungi even when blood cultures are negative.

Finally, histological examination allows us to observe the quantity and the morphological aspects of budding hyphae which can suggest a real overgrowth and a pathogenic role. More consideration needs to be given to selecting the appropriate antifungal agent for high-risk surgical patients.

## Consent

Written informed consent was obtained from patients for publication of these Case Reports and any accompanying images. A copy of the written consent is available for review by the Editor-in-Chief of this journal.

## Abbreviations

Candida spp.: *Candida* species; GMS: Gomori methenamine silver stain; PAS: Periodic acid Schiff stain.

## Competing interests

The authors declare that they have no competing interests.

## Authors’ contributions

PDC and GDV participated in the conception, design of the study and sequence alignment, and drafted the manuscript. DC carried out the histopathological studies. GG, FDA, GS, BS and GC participated in the clinical and surgical management. All the authors have read and approved the final manuscript.
